# Chronic Tenosynovitis due to *Mycobacteria kansasii* in an Immunocompetent Host

**DOI:** 10.1155/2018/3297531

**Published:** 2018-04-16

**Authors:** Michael S. Wang, Michael Berry, Alissa Lehto-Hoffman, Linh Vi, Nina Ramessar

**Affiliations:** ^1^Department of Medicine, Lakeland Health, St. Joseph, MI, USA; ^2^Osteopathic Medical Specialties, College of Osteopathic Medicine, Michigan State University, Lansing, MI, USA; ^3^Department of Surgery, Lakeland Health, St. Joseph, MI, USA; ^4^Infection Prevention, Lakeland Health, St. Joseph, MI, USA; ^5^Department of Pathology, Lakeland Health, St. Joseph, MI, USA

## Abstract

We report a case of a healthy 56-year-old male who presented with chronic swelling and erythema in his right hand. He had a prior chemical exposure several years ago and subsequent exposure to freshwater, fish tank, and soil. Laboratory data showed a slightly elevated CRP. An MRI was consistent with suggestive of flexor tenosynovitis. He underwent surgical debridement and was diagnosed with *Mycobacterium kansasii*. He was treated with clarithromycin, rifampin, and isoniazid, but subsequent susceptibility testing revealed resistance to isoniazid. Isoniazid was switched to ethambutol, but further susceptibility testing also suggested resistance to ethambutol. Antimicrobial therapy was stopped at 6 months due to clinical recovery, and the patient is currently doing well as of 6 months postdiscontinuation of therapy.

## 1. Introduction

*M. kansasii* is a nontuberculous atypical *mycobacterium* that typically causes respiratory infections [1, 2]. It has been found to be pathogenic in both immunocompromised and immunocompetent patients [1, 3, 4]. Nonrespiratory infections, including disseminated *M. kansasii*, have been well documented in the HIV population [3, 4]. Localized extrapulmonary disease in the immunocompetent population has been found to be relatively uncommon [5]. Here, we present a case of an otherwise healthy 56-year-old man who presented with a case of chronic *Mycobacterium kansasii* tenosynovitis. In this case, the patient had been previously diagnosed with contact dermatitis, treated over the span of several years.

## 2. Case Presentation

**Figure 1:**
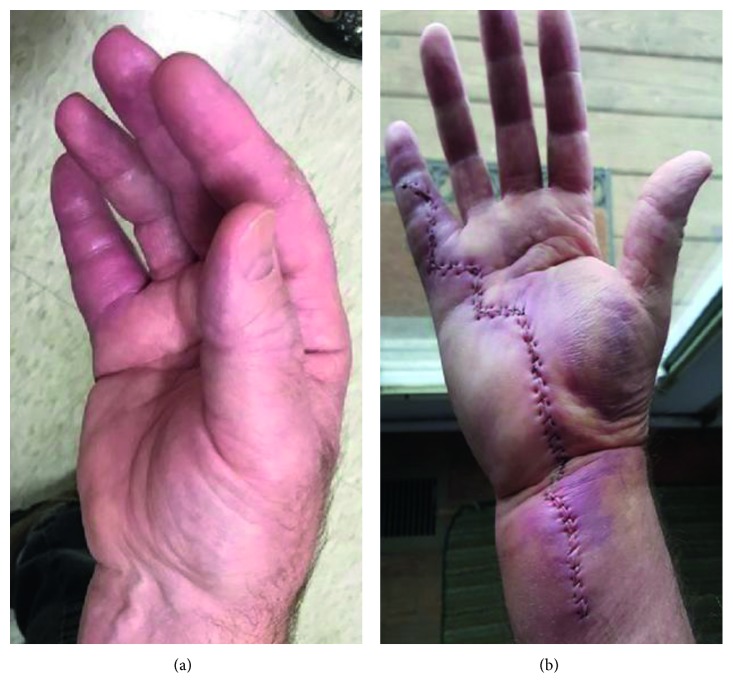
(a) Preoperative and (b) postoperative hand.

**Figure 2:**
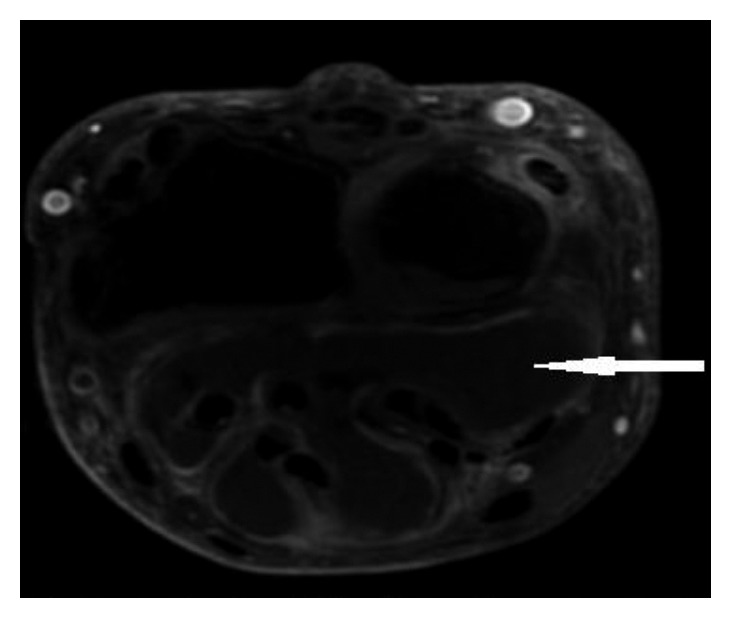
MRI wrist demonstrating tenosynovitis.

A 56-year-old man with no significant past medical history presented to the hand clinic with a 3-year history of right-hand pain and swelling. He works as an electrician and described a chemical spill on his right hand in 2013. He described the substance as consisting of hydraulic fluid, glycol, and penetrant. He was noted to have abnormal skin changes and intermittent swelling in his wrist and hand. At that time, he had been prescribed topical steroids without relief. He reported worsening of his right hand and wrist swelling over the past 2 months, shortly after helping his wife move a fish tank. He does not believe that his hand was in the water tank. He is frequently outdoors and works with the soil in his yard. He also frequently swims in a freshwater lake. His physical exam was abnormal for profound swelling of the right thumb, the volar aspect of the right wrist, and the right 5th finger (Figure 1(a)). Initial laboratory results showed a slightly elevated CRP of 1.4 mg/dL and normal ESR at 13 mm/h. An MRI demonstrated enhancement of the flexor tendon sheaths in the wrist and hand (Figure 2). He underwent a radical synovectomy of the hand and wrist (Figure 1(b)). He was initially started on intravenous piperacillin/tazobactam.

Pathology was suggestive of necrotizing granulomatous tenosynovitis. The initial piperacillin/tazobactam was discontinued. Given the patient's history of exposure to a fish tank, the biopsy tissue was sent for an acid-fast bacilli (AFB) culture. The AFB smear was positive. *Mycobacterium tuberculosis* was thought to be less likely, given the lack of tuberculosis exposure history and environmental exposures concerning for an atypical mycobacterial infection. He was initially started on rifampin and clarithromycin for possible *Mycobacterium marinum*. After the culture grew *Mycobacterium kansasii*, isoniazid (INH) was added. He improved on the above regimen. However, 4 months after starting the regimen, his susceptibilities via the agar proportion method suggested resistance to the INH. INH was discontinued, and he was started on ethambutol, in addition to the rifampin and clarithromycin. His isolate was then sent to an alternative reference lab, which tested resistance via the broth dilution method. This also demonstrated INH resistance. He completed 2 more months of this regimen for a total of 6 months of therapy. Shortly before completing the regimen, the second lab reported resistance to ethambutol. As he had clinically improved, the decision was made not to alter or extend therapy.

He has done well since discontinuing therapy, with resolution of the swelling and a return to his previous function.

## 3. Discussion

In both the HIV-positive and HIV-negative populations, the highest rates of *M. kansasii* infection were detected in an “inverted-T” geographic distribution that covered the southern and central United States. This endemic distribution suggests an environmental reservoir. However, *M. kansasii* has not been recovered from soil, has only rarely been detected in natural bodies of water, and has infrequently been cultured from potable water supplies [1, 5, 6]. We hypothesized that the patient's chemical injury to his hand likely provided an opportunity for the mycobacteria to penetrate his skin. Although his more recent potential exposure to a water tank led to testing for mycobacteria, we believe that his exposure to a freshwater lake was the more likely source of the infection.

Among cases of septic arthritis, *M. kansasii* most commonly presents as monoarticular synovitis, with arthrocentesis being diagnostic in less than 15% of cases [5]. *M. kansasii* tenosynovitis, although rare, has been documented in the medical literature [5, 7, 8]. It should be noted that of the reported nontuberculous mycobacteria causing tenosynovitis, *M. marinum* is the most common, followed by *M. kansasii*, *M. chelonei*, *M. terrae*, *M. fortuitum*, and *M. avium intracellulare* [9]. Tendon infections with nontuberculous mycobacteria often produce a strikingly similar clinical and histological presentation to tuberculous tenosynovitis [7, 8]. Nontuberculous mycobacterial tenosynovitis mainly presents as digital flexor tenosynovitis with chronic swelling and inability of the patient to make a full fist or flex the digits completely [7]. There may be no pain or fever, and the sedimentation rate (ESR) and C-reactive protein (CRP) may be normal [8]. Tissue biopsy is necessary for optimal diagnosis, and treatment typically includes both surgical debridement and antimycobacterial therapy [7–9].

In terms of treatment regimens, treatment recommendations for pulmonary *M. kansasii* infections had included INH, rifampin, and ethambutol [10, 11]. Susceptibility testing was previously recommended only for rifampin, as the susceptibility to other agents was often predicted by rifampin susceptibility [11]. In addition, treatment failures had previously been linked almost exclusively to rifampin resistance [12]. INH resistance has previously been reported in cases of prior INH exposure [11]. Macrolides such as clarithromycin and the fourth generation fluoroquinolone moxifloxacin have demonstrated very good *in vitro* activity against *M. kansasii* and may be an alternative to INH [10].

As noted, resistance testing was initially performed via the agar proportion method and subsequently followed by the broth dilution method. The isolate in this case did not conform to previous findings, as the isolate was susceptible to rifampin but resistant to INH in two reference labs. Our patient also had no prior exposure to INH. The reason for different susceptibility results of ethambutol between the two reference labs is unclear. *M. kansasii* susceptibility testing has previously been performed via both the agar proportion method and broth dilution method [12–14]. A previous study observed susceptibility results in *M. tuberculosis* testing found broth dilution to produce an MIC closer to the true MIC [15]. This was thought to be from a lower degree of absorption and degradation in the liquid medium, as opposed to the agar method [15].

## 4. Conclusion

Our patient with atypical mycobacteria was successfully treated with surgical debridement and 6 months of antimycobacterial therapy, with 6 months of 2-drug therapy consisting of clarithromycin and rifampin. He had also received 4 months of INH and 2 months of ethambutol, which may not have significantly contributed to his resolution.
